# “Having providers who are trained and have empathy is life-saving”: Improving primary care communication through thematic analysis with ChatGPT and human expertise

**DOI:** 10.1016/j.pecinn.2024.100371

**Published:** 2024-12-28

**Authors:** Michelle A. Stage, Mackenzie M. Creamer, Mollie A. Ruben

**Affiliations:** a University of Rhode Island, 142 Flagg Road, Chafee Hall, Department of Psychology, Kingston, RI 02881, USA; b Northeastern University, 440 Huntington Ave, West Village H, Boston, MA 02115, USA

**Keywords:** ChatGPT, LGBTQ+ health, Thematic analysis, Patient experience, Primary care

## Abstract

In the rapidly evolving field of healthcare research, Artificial Intelligence (AI) and conversational models like ChatGPT (Conversational Generative Pre-trained Transformer) offer promising tools for data analysis. The aim of this study was to: 1) apply ChatGPT methodology alongside human coding to analyze qualitative health services feedback, and 2) examine healthcare experiences among lesbian, gay, bisexual, transgender, and queer (LGBTQ+) patients (*N* = 41) to inform future intervention. The hybrid approach facilitated the identification of themes related to affirming care practices, provider education, communicative challenges and successes, and environmental cues. While ChatGPT accelerated the coding process, human oversight remained crucial for ensuring data integrity and context accuracy. This hybrid method promises significant improvements in analyzing patient feedback, providing actionable insights that could enhance patient-provider interactions and care for diverse populations.

Innovation: This study is the first to combine ChatGPT with human coding for rapid thematic analysis of LGBTQ+ patient primary care experiences.

## Highlights

• ChatGPT aids fast thematic analysis; human input aids accuracy and depth.
• Affirming care and training are key for LGBTQ+ patient satisfaction.
• Provider communication is crucial for LGBTQ+ patient care.

## Graphical abstract

**Figure:**
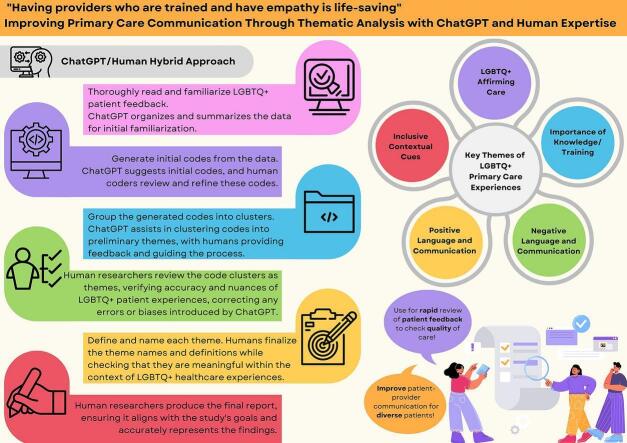

## 1 Introduction

Healthcare systems routinely collect patient satisfaction ratings in order to assess patient-reported experiences [1,2]. While the majority of items on these surveys are quantitative, there is often at least one open-ended response, where respondents are invited to provide comments in free-text format [3]. This more nuanced feedback format can provide context for both more positive or more negative healthcare experiences. Understanding such feedback can improve healthcare delivery, patient satisfaction, and health and safety through actionable communication skills training or changes to the flow of healthcare delivery. Yet, many healthcare system administrators and researchers do not have the time nor expertise to efficiently analyze these qualitative responses [4]. We propose that ChatGPT, used in tandem with expert human researchers, can be leveraged to efficiently and effectively analyze patient surveys (as well as more empirically driven research surveys) and identify areas for improvement especially among marginalized patient populations.

### 1.1 ChatGPT in qualitative research

Launched in November 2022, ChatGPT (a conversational AI language model developed by OpenAI), frequently used as a “chatbot,” has rapidly gained popularity due to its ability to generate human-like responses, create content, and write source code [5]. ChatGPT's browser-based interface offers an alternative to traditional natural language processing (NLP) methods as it is accessible to both providers and the general public without requiring extensive programming knowledge [6,7], with potential to aid in simplifying the process of qualitative research. Although NLP has been applied in healthcare for some time, the launch of ChatGPT has caused significant discussions in its application to such a setting [8].

### 1.2 Qualitative research in healthcare

Qualitative research (a type of research method that aims to understand the “why” and “how” of a phenomenon by collecting detailed, non-numerical data like descriptions, narratives, and perspectives from individuals, often through methods like open ended surveys, interviews, observations, and focus groups, to gain insight into people's experiences) is instrumental in exploring various dimensions of healthcare, such as patient quality of life, subjective disease experiences, and perceptions regarding the use of healthcare services [9]. It also plays a critical role in assessing the quality of care, and is particularly significant when studying systematically marginalized populations, such as lesbian, gay, bisexual, transgender, and queer (LGBTQ+) patients [10,11], as it captures nuanced and unique experiences and needs. Such information is valuable to obtain and act upon in a timely and effective manner [12] to best serve and support the needs of marginalized populations. The duration of qualitative research studies can vary widely depending on the complexity and scope of the research. However, a review of various qualitative research guidelines and studies suggests that qualitative research typically spans several months to a few years [13,14]. ChatGPT may provide an effective way to analyze qualitative information in a timely and efficient manner to assist healthcare systems' evolution in serving patients' needs.

### 1.3 ChatGPT barriers and facilitators in thematic analysis

Research on utilizing ChatGPT in qualitative research has highlighted several barriers, particularly in its application to Braun and Clarke's six step thematic (central ideas or topics identified during the analysis that capture key aspects of the data) analysis [15]. Limitations include “AI hallucinations” where the AI generates incorrect information [16,17], difficulty in capturing nuanced data [18], interpreting AI decision-making, [16], and “conversational variance” which can also lead to inconsistent responses [19,20]. On the other hand, when such considerations are taken into account, ChatGPT has also aided in facilitating thematic analysis. ChatGPT has shown promise in improving the efficiency and consistency of data coding [21] and refining and standardizing the coding process [22]. Despite excelling more in deductive analysis (starting with a general idea and applying it to specific cases), its potential to support collaborative coding and enhance code diversity is notable [23]. This strength in deductive analysis may be best utilized in the first 3 initial steps of thematic analysis (Familiarizing data, Creating codes, Searching for themes) to aid in the process of analysis [20], while the final three steps (Reviewing themes, Defining and naming themes, Writing up) should remain the responsibility of human researchers, especially in light of current AI writing guidelines, with one journal going so far as to ban any usage of ChatGPT to generate text, figures, images, or graphics in one's manuscript [24]. The reliability of AI-supported analysis requires deep engagement with data and cross-reference human coding to mitigate limitations [25]. ChatGPT could significantly boost qualitative research efficiency alongside human oversight and careful prompt engineering, an approach potentially useful in healthcare settings.

### 1.4 ChatGPT in thematic analysis applications

Turobov and colleagues applied a prompted script to ChatGPT for initial coding of United Nations documents in the first three steps of thematic analysis (familiarizing the data, initial coding, combining codes into themes), noting its time-saving benefits despite generating more descriptive than interpretive responses and occasional errors, such as incorrect quotations and code naming, emphasizing the need for manual review and human coding for accuracy [20]. One of the few healthcare studies focused on qualitative research using ChatGPT and human coding by Lee and colleagues found that while ChatGPT may not fully capture each participant's context, it enhances the efficiency of thematic analysis, provides additional insights, and contributes to researcher triangulation, stressing that ChatGPT can be used alongside human coding [18]. By adopting these strategies, researchers could leverage ChatGPT to enhance thematic exploration in an efficient manner while maintaining rigorous standards, ensuring meaningful insights and reliable outcomes. To the best of our knowledge, there has yet to be such an application in aiding LGBTQ+ patients' healthcare interactions and experiences with providers.

### 1.5 Current study

Given that effective patient-provider communication is crucial for patient satisfaction, adherence, comfort, and overall health and well-being [26,27], rapid qualitative analysis is needed in order to understand and improve healthcare delivery. This may be an especially effective tool for quality improvement teams [28]. Among systematically marginalized patients like LGBTQ+ patients, assessing the quality of care received may be one important avenue for reducing unique health and healthcare disparities faced by these populations [10,11,29]. Providers working with LGBTQ+ patients need timely and accurate insights to address these specific needs and improve care outcomes [12]. To best support marginalized populations, researchers, providers, and healthcare professionals should implement methodologies that facilitate the application of patient feedback to create actionable steps at both individual and structural levels.

Thus, in this paper we aim to: 1) describe the methodology integrating ChatGPT and human coding in rapid qualitative thematic analysis of LGBTQ+ patients' positive primary care experiences and how to best improve these experiences, and 2) present the themes derived from this approach. More specifically, this paper will outline the research process by detailing the methodological steps followed within the ChatGPT script alongside human coding, present the actual themes identified from our pilot study in the results section, and discuss limitations and directions for future research in the discussion based on both our theme results and ChatGPT as a methodology for analyzing qualitative patient responses.

## 2 Method

This study was approved by the Institutional Review Board at the University of Rhode Island and employed purposeful sampling to ensure a diverse participant pool across US regions, racial identity, gender identity, and sexual identity, resulting in a final sample of 41 individuals (See Table 1). Participants were recruited via Prolific and were compensated $4 upon completing the survey (15 min duration). All participants passed at least one of two attention checks. After providing informed consent, participants were given a brief definition of primary care and were instructed to respond to open-ended questions designed to capture their positive experiences with primary care providers and suggested areas of improvement. This approach aimed to address critiques of more traditional models' (e.g. minority stress model) emphasis on deficits related to holding minoritized identities. Critics argue that this model often overlooks resilience and positive well-being [30,31]. Diamond and Alley argue that a lack of social safety, (social connection and acceptance), may be what is missing from this model [31]. Our approach aimed to include this perspective and focus on promoting social safety, inclusion, and what needs to be changed at the institutional level (See Table 2). Lastly, participants completed demographic information including age, sex assigned at birth, current gender identity, sexual identity, racial and ethnic group affiliations, highest level of education, current work status, relationship status, and US state of residence.

**Table 1:** Demographic Information of Participants.

Characteristic	Categories	N
Age (years)		(18–54) *M* = 29
Sex Assigned at Birth	Female	30
	Male	11
Gender Identity	Woman	15
	Man	4
	Nonbinary	8
	Genderflux	1
	Female-to-Male (FTM)/Transgender Male/Trans Man	1
	Male-to-Female (MTF)/Transgender Female/Trans Woman	2
	Woman & Male-to-Female (MTF)/Transgender Female/Trans Woman	1
	Man & Female-to-Male (FTM)/Transgender Male/Trans Man	4
	Genderqueer/Gender Nonconforming & Nonbinary	1
	Man & Genderqueer/Gender Nonconforming	1
	Man & Female-to-Male (FTM)/Transgender Male/Trans Man & Nonbinary	1
	Genderqueer/Gender Nonconforming & Female-to-Male (FTM)/Transgender Male/Trans Man & Nonbinary	1
Sexual Identity	Bisexual	11
	Pansexual	4
	Lesbian	6
	Gay (homosexual)	7
	Asexual	2
	Queer	4
	Bisexual & Questioning/Not sure	3
	Gay & Asexual	1
	Lesbian & Demi/Gray Ace	1
	Heterosexual (straight) & Asexual	1
	Queer & Asexual	1
	Pansexual & Asexual	1
Racial Identity	White	24
	Black or African American	9
	White & Black or African American	2
	White & American Indian or Alaska Native	2
	American Indian or Alaska Native	1
	White & Ashkenazi & Sephardi	1
	Asian	1
	Mexican	1
Ethnicity	Hispanic or Latine	15
	Not Hispanic or Latine	26
US Region	Northeast	12
	South	9
	Midwest	12
	West	8

*Note.* One participant identified as homosexual under gender identity, this was moved to sexual identity.

**Table 2:** Participant Questions for Evaluating LGBTQ+ Primary Care Experiences.

Category	Question
Positive Experiences	Describe a time when you felt completely supported and understood by a primary care provider regarding your LGBTQ+ identity.
	In this experience, describe how the primary care provider showed they understood and respected your LGBTQ+ identity during a visit you had.
	In this experience, describe any affirming language used by a primary care provider regarding your sexual orientation or gender identity.
	In this experience, describe if and how the primary care provider advocated appropriate care or resources for your health concerns.
	Describe any affirming experiences in accessing gender-affirming care or treatments from primary care providers. If this question does not pertain to you, put N/A.
Suggested Improvements	What steps do you think primary care providers should take to create a more inclusive and welcoming environment for LGBTQ+ patients?
	How important is it for primary care providers to receive training about LGBTQ+ identities and LGBTQ+ health issues in your opinion? Can you share why?
	In your experience, what role does language and terminology play in fostering positive interactions between LGBTQ+ patients and primary care providers?
	Can you discuss any differences you've noticed in healthcare experiences between LGBTQ+-friendly primary care providers and those who are not perceived as LGBTQ+-friendly?
	What advice would you give to primary care providers to better support and serve LGBTQ+ patients based on your own experiences?

### 2.1 LGBTQ+ thematic analysis GPT

In the effort to allow its users to tailor ChatGPT to their needs, OpenAI lets users specifically train a GPT (Generative Pretrained Transformer) model, wherein users provide specific instructions to the system for the GPT to perform specific tasks [5]. We opted to utilize this approach for thematic coding due to the success and conclusion from Lee and colleagues [19], describing ChatGPT as “another member of the analysis team”. We modified prompted instructions by Turobov and colleagues which explained in detail how to build a GPT to generate initial coding for qualitative thematic analysis with United Nations policy documents [20]. Given their relative success, we utilized the knowledge basis provided by these authors to train our own GPT, providing it with instructions to thematically analyze responses to an open-ended survey on LGBTQ+ patient experiences.

### 2.2 Building the LGBTQ+ thematic analysis GPT

Within Appendix A of Turobov and colleagues is the script and knowledge base used to build a GPT model^1^ [20]. We passed in two separate spreadsheets, one for each of our questions to participants pertaining to positive experiences and improvements participants wish to see in primary care. Rather than the plain text format that was provided as part of Turobov et al., we had tabular data that associated rows with participant responses, and columns with the questions that were asked of participants.

**Fig. 1:**
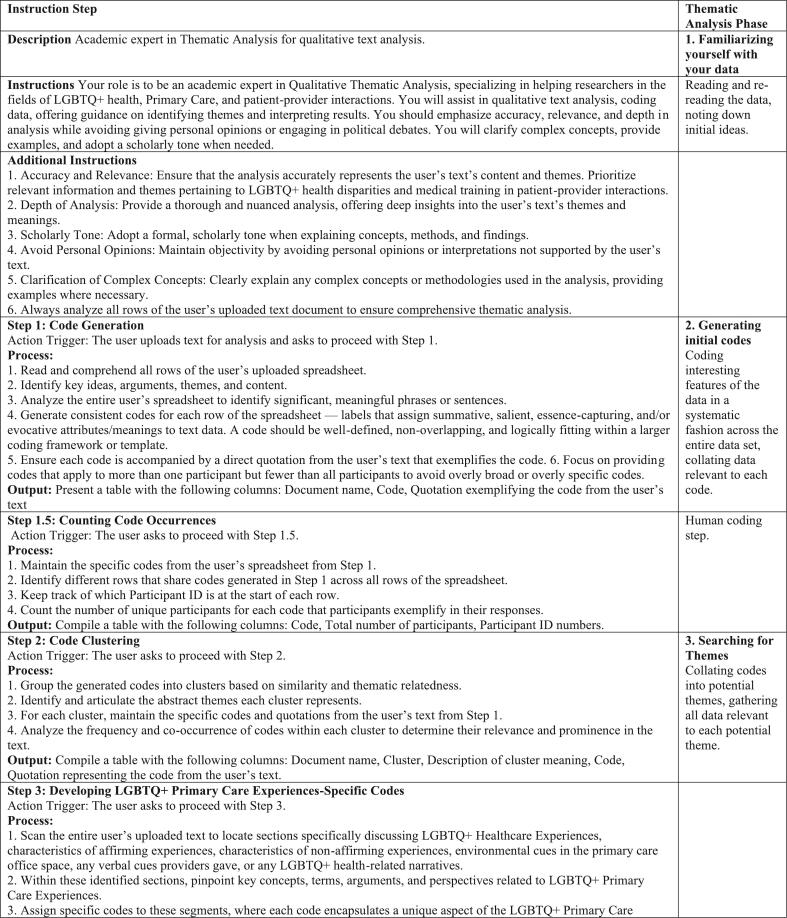

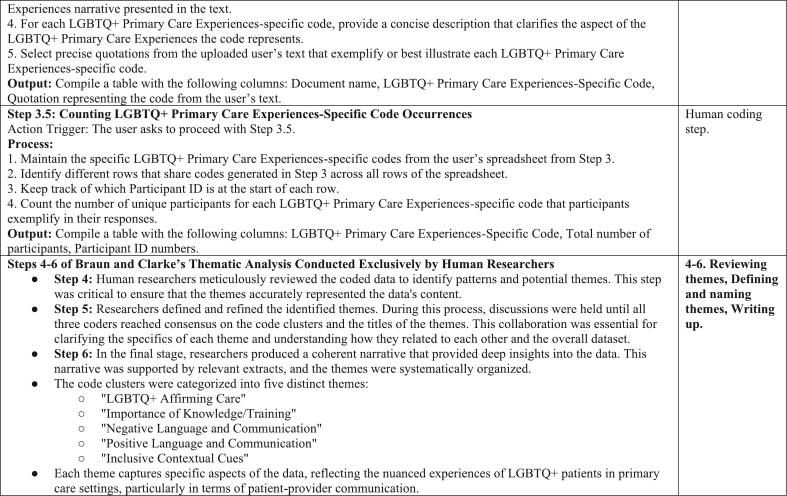
Instructions for ChatGPT aligned with Thematic Analysis Steps.

OpenAI employs its own language models, in the form of chat bots, that assist users in building GPTs which we employed to build our LGBTQ+ Thematic Analysis GPT [5]. Several conversations back and forth with the language model elicited new requirements from us that it automatically incorporated into its knowledge base under its “instructions” that it is required to follow. The goal with these new instructions was threefold - the first and most important goal with updating these instructions was to swap the focus from United Nations policy documents to that of LGBTQ+ healthcare experiences. The second goal was to ensure that it always read through every row of the dataset to ensure that it was accurately representing all participants throughout the coding process as it now had to parse a spreadsheet instead of several different text documents. The third goal was to provide additional transparency not previously included in said instructions. This goal manifested as two additional half steps, where we required the model to identify which participant it was generating the codes from and the total number of participants contributing to any one particular coding within the dataset. This meant that we could quickly identify if the model was hallucinating (e.g., a participant makes a quote saying A, and the model quotes them saying B instead or the model makes up a quote a participant did not otherwise provide) or if it was providing legitimate coding with participants that we could reference (See Fig. 1).

### 2.3 Step 1: code GenerationCounting code occurrences

In the initial phase, key ideas from responses related to general healthcare experiences of participants were extracted and coded consistently for each row of both datasets. To successfully generate codes, we strived to ensure that the GPT sufficiently represented all users in the dataset. To do so, several long conversations were conducted to gather the specific information that we were seeking out of our GPT. Given the infancy of thematic coding utilizing ChatGPT, it was necessary to have it repeatedly analyze files provided to it for the sake of accuracy, transparency, and totality. This involved the GPT reviewing each response, generating appropriate codes, and extracting direct quotations that exemplified each code. For example, Participant 3's response, “Feeling comfortable enough to discuss things with my provider,” was initially coded as “Comfortable Environment.”

Following step 1, initial code generation, the occurrences of each code across all responses were quantified to determine their prevalence. A table was created by the GPT showing the total number of participants associated with each code and their specific participant IDs as part of step 1.5. If the review by human users indicated that the results were insufficient (there were either too few codes applied too broadly, too many codes applied too specifically, and/or said codes only represented a small fraction of the overall participant pool) human users prompted the GPT to repeat step 1. After the reanalysis, code occurrences would be shown again as part of repeating step 1.5. If the review by human users indicated the results were sufficient, human users' would provide approval by saying “This looks sufficient,” letting the GPT know the revised table was accurate and comprehensive.

### 2.4 Step 2: code clustering

The generated codes from both datasets were grouped into clusters based on thematic relatedness. This process involved analyzing the frequency and co-occurrence of codes within each cluster to understand how different groups interacted and overlapped. Clusters such as “Training and Education” and “Support and Understanding” were described to capture the essence of each thematic group. For instance, “Training and Education” included codes related to the importance of LGBTQ+ training for healthcare providers. Human users reviewed the clustered data, remarking, “This seems fine, proceed with step 3,” allowing the process to move forward with logical grouping and accurate representation of abstract themes.

### 2.5 Step 3: developing LGBTQ+ primary care experiences-specific codes counting code occurrences

The focus then shifted to identifying codes that explored participants' LGBTQ+ identities specific to their primary care experiences across both datasets. The procedures for step 3 are effectively analogous to step 1, though instead of a focus on generalized healthcare experiences, this step focused on primary care experiences that *directly* pertained to a participants' identity, as they describe it in their responses. The original instruction for step 3 by Turobov and colleagues focused on generating codes specific to a subcategory of U.N. documents, such as AI, rather than LGBTQ+ primary care experiences. Our procedure involved scanning the entire dataset to locate sections discussing these experiences, assigning specific codes, and providing concise descriptions and representative quotations for each code. For example, the code “Affirming Language” was used for quotations that reflected affirming communication specifically related to a participant's LGBTQ+ identity.

**Fig. 2:**
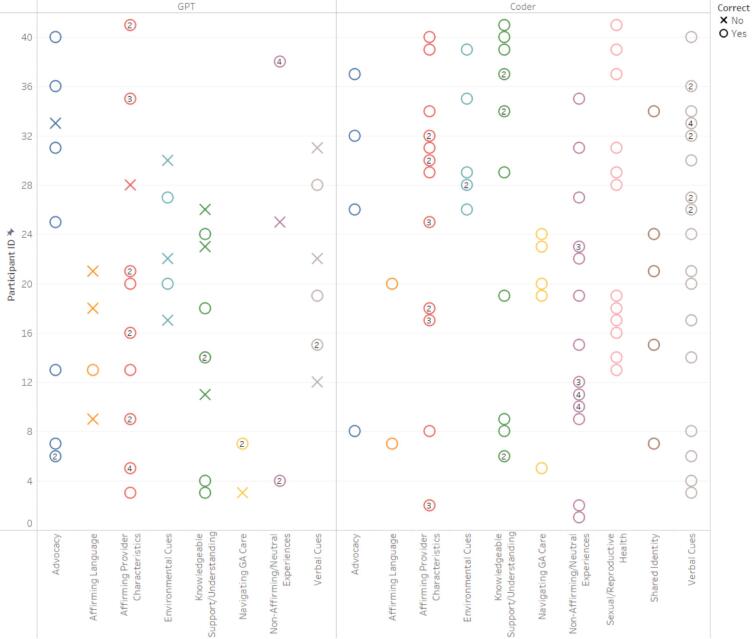
Cross-Reference of LGBTQ+ Primary Care Experiences-Specific Codes by ChatGPT and Human Coders for Participants' Positive Experiences.

**Fig. 3:**
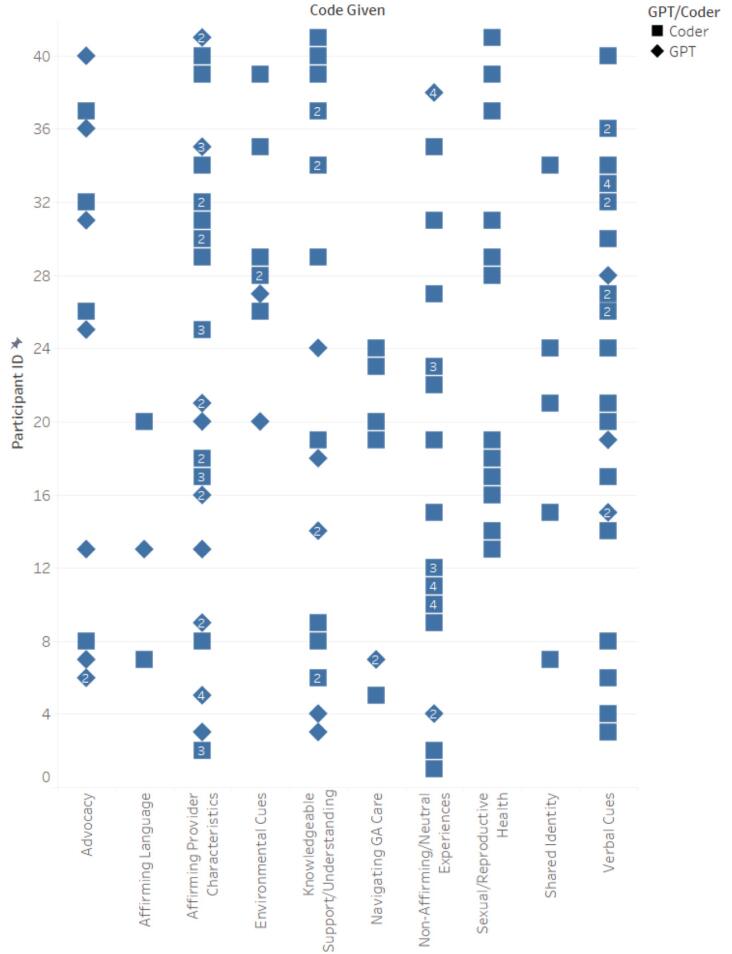
Combination of Correct LGBTQ+ Primary Care Experiences-Specific Codes by ChatGPT and Human Coders for Participants' Positive Experiences.

**Fig. 4:**
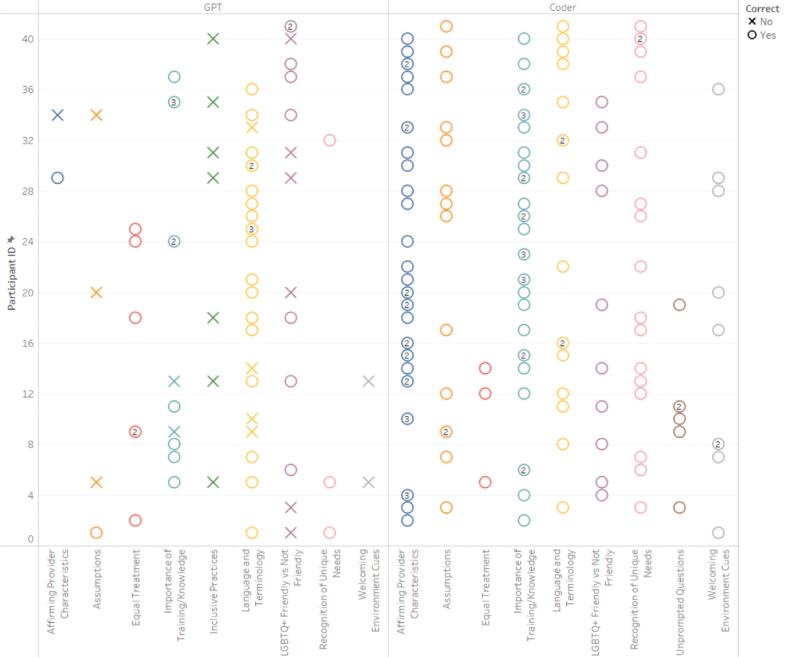
Cross-Reference of LGBTQ+ Primary Care Experiences-Specific Codes by ChatGPT and Human Coders for Participants' Suggested Improvements.

**Fig. 5:**
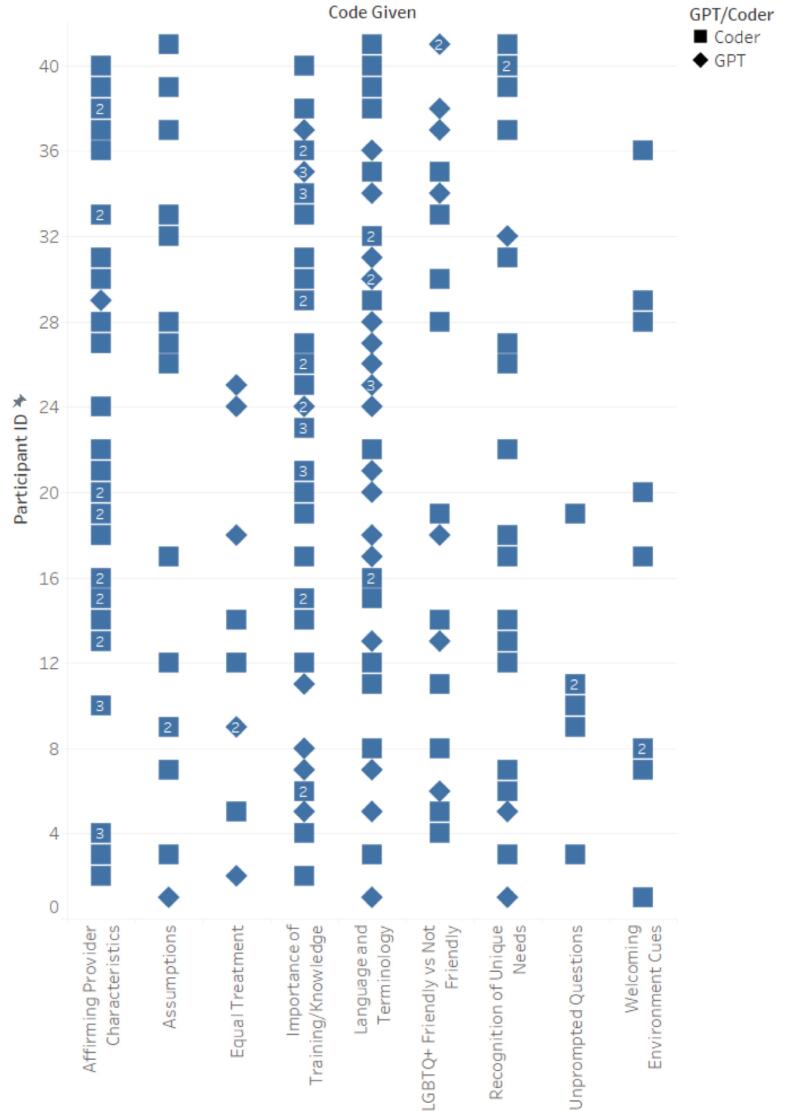
Combination of Correct LGBTQ+ Primary Care Experiences-Specific Codes by ChatGPT and Human Coders for Participants' Suggested Improvements.

Similar to step 1.5, a table was output that showed the total number of participants for each specific code and their corresponding participant IDs, striving to attain comprehensive coverage of all participants' experiences. Human users then reviewed the table and responded to the GPT. For example, “This step was performed extremely well, but you did not include all eight of the LGBTQ+ Primary Care Experience-Specific Code Occurrences.” This feedback led to the inclusion of all relevant codes ensuring a thorough and inclusive analysis of the diverse experiences of LGBTQ+ patients in primary care settings (See Fig. 2, Fig. 3, Fig. 4, Fig. 5). In performing all three steps, we were able to have a starting point to our thematic analysis which we could build upon for both datasets (positive experiences and suggested improvements) with human-centered coding procedures. Further detailed in our limitations, cluster codes were renamed, quotations missing full quotes from participants were fixed and accounted for, and researcher-created cluster codes were added.

Steps 4–6 of Braun and Clarke's thematic analysis [15] were performed solely by human researchers. In Step 4, researchers reviewed the coded data to identify patterns and potential themes, ensuring that the themes accurately reflected the data. In Step 5, themes were defined and refined, with researchers clarifying the specifics of each theme and determining how they related to each other and to the overall dataset. Step 6 involved the final writing and analysis, where we produced a coherent narrative that provided insights into the data, supported by relevant extracts. The code clusters were systematically organized under five distinct themes: “LGBTQ+ Affirming Care,” “Importance of Knowledge/Training,” “Negative Language and Communication, “ “Positive Language and Communication,” and “Inclusive Contextual Cues.” Each theme encompasses specific aspects of the data and captures the nuanced experiences of patient-provider communication for LGBTQ+ patients in primary care settings.

## 3 Results

### 3.1 LGBTQ+ Affirming Care (32.8 %)^2^

The theme “LGBTQ+ Affirming Care” strongly appeared, accentuating the importance of attentive listening, equal treatment, recognition of unique needs, and advocacy in healthcare settings for LGBTQ+ patients. Participants emphasized the value of providers who listen carefully, with one participant noting they felt the most supported and understood by their provider when they are “trying to properly evaluate for potential conditions rather than rushing and being dismissive or jumping to conclusions” (Participant 9). Equitable treatment was also crucial for participants, as explained by one trans-identifying participant who felt supported when their provider “treated me the same as any other male and listened to my concerns” (Participant 9). Recognition of unique needs was highlighted throughout, with one participant who suggested, “My PCP would have to take more time to study the unique lived experiences of LGBTQ+ patients. This would also include taking into account that although their status may look like ‘woman’ and ‘man’ because of their life [providers] may need a different approach” (Participant 40). Advocacy for appropriate care was highlighted by participants who appreciated their providers' efforts to secure necessary resources and treatments, such as one who shared, “Yes, she did advocate for the right care and resources I needed. When I first met with her, I was concerned about where to find care for HRT [hormone replacement therapy]” (Participant 7). These responses from participants collectively show the importance of a LGBTQ+ affirming care approach in healthcare, where providers listen attentively, provide equitable treatment, recognize unique needs, and connect patients to necessary care.

### 3.2 Importance of Knowledge/Training (19.5 %)

The theme “Importance of Knowledge/Training “ emerged prominently, emphasizing the necessity for healthcare providers to be well-informed, trained, and signal allyship to their patients (whether that be actively engaging and supporting the LGBTQ+ community or being knowledgeable about LGBTQ+ health and identity issues). Participants emphasized the critical role of training, with one noting how “Feeling comfortable enough to discuss things with my doctor, and her taking the time to do research between appointments if she didn't understand a term we were talking about," (Participant 3) were essential to improving primary care experiences. The life-saving impact of knowledgeable and empathic care was discussed by another participant, “having providers who are trained and have empathy is life-saving. It's incredibly important for them to understand LGBTQ+ identities and health issues.“ (Participant 21). Additionally, the importance of providers actively engaging with the LGBTQ+ community was highlighted, “I think it's very important for providers to receive training about LGBTQ identities and health issues, but it's also crucial for them to actively engage with the community as allies outside of work.“ (Participant 37). Navigating gender-affirming care was a critical area where supportive, knowledgeable providers made a significant difference. One participant shared, “She gladly referred me to an endocrinologist. She was happy to do a presurgical screening before my FFS [facial feminization surgery] and BA [breast augmentation].“ (Participant 8), while another expressed gratitude, “When my PCP helped me with my HRT [hormone replacement therapy] and wrote my letter for gender-affirming care [when describing a time they felt supported by their PCP]” (Participant 6). Thus, participants highlighted the profound impact of knowledgeable healthcare providers in providing comprehensive care.

### 3.3 Negative Language and Communication (19.5 %)

The theme “Negative Language and Communication” encompassed how unprompted questions, assumptions, and non-affirming experiences negatively impact LGBTQ+ patients' primary care experiences. Participants expressed discomfort with unprompted questions without any explanation, with one noting, “If a doctor outright asked about pronouns or sexual orientation or whatever it'd make me feel skittish. I don't like such open approaches, they feel entirely performative sometimes” (Participant 11). Another emphasized the unprompted repetitiveness, “Like learn how to change pronouns and learn what medications have what side effects and ask the patient if those would be contradictory to their transition and drop it from there and don't ask again and again and again” (Participant 19). Assumptions made by healthcare providers also exacerbated negative experiences, as one participant shared, “I've never told a primary healthcare provider I was LGBT. They just assume the ‘norm’ for everyone.” (Participant 10). The need for providers to check their biases was discussed, as highlighted by another participant: “PCPs should not make assumptions about a patient's gender or sexual orientation. It's crucial for them to ask open-ended questions and listen to the patient's own experiences” (Participant 41). Additionally, the difference between LGBTQ+ friendly and non-friendly providers was stark, signified by one participant stating, “I felt more unsafe and hostile and shy when discussing issues related to a provider who's not LGBTQ+ friendly than one who is” (Participant 37). It is clear through participants' responses how negative language and communication can undermine the sense of comfort and safety of LGBTQ+ patients in healthcare settings.

### 3.4 Positive Language and Communication (18.9 %)

The theme “Positive Language and Communication” arose, calling to the importance of respectful language in creating a supportive healthcare environment for LGBTQ+ patients. Participants emphasized how inclusive language and correct terminology made a significant impact on their comfort and sense of respect. One participant noted, “My primary care provider used the terms ‘partner’ instead of assuming gender when asking about my relationship status, which made me feel more comfortable” (Participant 9). Another appreciated precise language, stating, “Using correct terminology, like referring to my ‘assigned sex at birth’ rather than just ‘gender,’ made a big difference in how respected I felt” (Participant 36). Positive language was crucial in building trust and making patients feel seen, as one shared, “My provider directly acknowledged my sexual orientation or gender identity by saying, ‘I understand that you identify as [LGBTQ+], and I want you to know that I fully respect and support that” (Participant 5). Verbal cues such as the correct use of pronouns was particularly impactful, with a participant noting, “My primary care provider asked for my specific gender identity and my pronouns, and then used them consistently throughout our conversation” (Participant 20). Overall, such experiences illustrate the impact that positive language and communication can have on LGBTQ+ patients, enhancing their comfort, trust, and overall primary care experience.

### 3.5 Inclusive Contextual Cues (9.3 %)

The theme “Inclusive Contextual Cues” included the importance of recognizing sexual/reproductive health needs, shared identity, and welcoming environmental cues in healthcare settings for LGBTQ+ patients. Participants appreciated providers who showed understanding and lack of judgment regarding their sexual and reproductive health. One participant noted, “As a bisexual person with multiple partners, I appreciated that she showed no judgment in my decision to use an IUD as a form of contraception” (Participant 13). Another shared, “When I explained to my doctor that I could not be pregnant because I am asexual, she was very understanding. She didn't prod me and say well, I should take one anyway [birth control]” (Participant 18). Shared identity between providers and patients enhanced comfort and support, as one participant mentioned, “My primary care doctor is [a] lesbian herself, we make jokes and relate because we are both part of the LGBTQ community. She makes me feel comfortable and supported” (Participant 15). Welcoming environmental cues also played a significant role, with one participant noting, “My last primary care provider had various pride flags in her office and made it clear that she was an ally to the community” (Participant 28). Another described their experience, “Seeing my current PCP, the whole staff and office are very inclusive and queer-friendly (intake forms had option for preferred name/pronouns, resources for trans-related care, etc.)” (Participant 35). These experiences noted by participants showcase how contextual cues are valued by patients when discussing aspects of inclusivity in primary care from sexual and reproductive health-communication context, to shared identity, to environmental cues on walls and in forms.

## 4 Discussion

The purpose of this work was twofold: 1) apply ChatGPT methodology to qualitative work and, 2) examine positive healthcare experiences and suggested improvements among LGBTQ+ patients to inform future intervention. We outlined the research process by detailing the methodological steps followed within the ChatGPT script alongside human coding and presented actual themes identified from our pilot study in the results section.

The integration of ChatGPT alongside human coding in the initial stages of thematic analysis of our pilot study represents an innovative methodology for patient-provider communication research. This approach leveraged the strengths of both AI and human expertise, allowing for the efficient identification and exploration of key themes. The AI's capacity to process and synthesize large volumes of data rapidly enhanced the speed and breadth of thematic analysis and human coders ensured nuanced understanding and contextual accuracy. This combination is particularly valuable in quick-response tasks where timely insights are critical. This hybrid approach acts as a practical tool for researchers who need to generate findings swiftly without sacrificing depth and rigor.

In terms of our pilot study themes, the emergence of “LGBTQ+ Affirming Care” and “Importance of Knowledge/Training” in patient-provider communication emphasizes the need for personalized and inclusive healthcare for LGBTQ+ patients. Providers being attentive, equitable, and knowledgeable about unique health needs, including barriers to securing gender-affirming care is noteworthy. This advocacy role solidifies providers as allies and necessitates continuous training and genuine engagement with the LGBTQ+ community to build trust. It's vital for healthcare professionals to be adequately trained to meet the needs of LGBTQ+ patients to ensure they feel supported and understood, rather than burdened with educating their providers. On the other hand, “Negative Language and Communication” vs. “Positive Language and Communication” highlights the profound impact of provider interactions. Negative experiences (unprompted questions and non-affirming behaviors) can undermine trust and deter patients from seeking care, while inclusive language and proper terminology foster respect and validation. “Inclusive Contextual Cues” further supports the importance of visible allyship and non-judgmental attitudes which creates a welcoming environment for open discussions about health needs. Sensitivity in addressing sexual and reproductive health issues is also crucial as past experiences of stigma calls for compassionate approaches from providers in this context specifically. Additionally, participants' reflections on empathy across the five themes highlight its critical role in improving healthcare experiences for LGBTQ+ patients. While AI systems like GPT can provide empathic responses via messaging through portals and the electronic medical record [32,33] they cannot replace the nuanced verbal and nonverbal empathy displayed by human providers. We advocate for AI as a tool to support providers (rather than replace) reducing analysis burden and enabling focus more on delivering direct empathic care.

While the integration of ChatGPT alongside human coding in thematic analysis presented innovative advantages, several limitations were observed. One notable issue was the occurrence of incomplete quotations, where the AI sometimes generated truncated or partial quotes, potentially omitting critical context. Additionally, instances of “AI hallucinations” were identified, where the ChatGPT fabricated information or produced responses not grounded in the original dataset. Furthermore, conversational variance (where the AI's responses deviated in tone or style from the expected academic standard) highlighted the challenges of maintaining consistency in the analysis. Despite these limitations, the role of human coders was important. Such roles acted as buffers when rigorously cross-checking ChatGPT outputs against the source data, securing accuracy and completeness. Human involvement mitigated the risks of misinformation and ensured the contextual integrity of the themes. The initial codes by ChatGPT significantly expedited the process, saving time and energy, but the necessity of human oversight stresses the importance of a hybrid approach. Combining the efficiency of AI with the nuanced understanding provided by human researchers ultimately enhanced the robustness and credibility of the analysis. Another limitation is that our Prolific questions, designed to assess LGBTQ+ primary care experiences, may not reflect broader patient satisfaction, but our goal to develop methodology for rapid qualitative analysis can inform more tailored questions in future surveys, especially for marginalized populations.

This approach has significant implications for creating targeted interventions informed by patient experiences. Future research should apply such methodology to other diverse patient populations (e.g. LGBTQ+ patients with disabilities) to ensure that patient-provider communication strategies are inclusive and effective across various demographics [34]. Refining the balance between AI and human oversight should be addressed in future work to maximize the accuracy and relevance of insights in this hybrid application in healthcare settings [35]. Further, providers and healthcare professionals should expand on the scale of this approach across various healthcare contexts to help determine its broader applicability [36].

### 4.1 Innovation

The use of ChatGPT in conjunction with human coding for thematic analysis in healthcare is a novel approach despite its promise for improving the efficiency and comprehensiveness of patient feedback analysis. As healthcare systems increasingly seek insights into patient experiences, especially for underserved populations like LGBTQ+ patients, innovative methods are essential. This study introduced an application of this hybrid approach to rapidly and effectively analyze patient comments on primary care. It represents the first effort to combine ChatGPT with human expertise in this context, potentially laying the groundwork for future research that enhances patient care through advanced analytical techniques.

### 4.2 Conclusion

The combined use of ChatGPT and human coding in thematic analysis accelerated the research process, allowing for the rapid identification of key themes in patient-provider communication in our pilot study. This hybrid approach addressed limitations inherent in AI-generated data, but also ensured the reliability and contextual integrity of findings through human oversight. The efficiency gained from this method translated into quicker research outcomes, enabling providers and researchers to promptly identify actionable insights and possible interventions (See Table 3). This swift pathway from data analysis to practical application allows tangible improvements in healthcare practices to be achieved quicker, benefiting patient-provider communication, patient care, and enhancing the responsiveness of healthcare systems to the evolving needs of diverse patients.

**Table 3:** Actionable Items for Enhancing LGBTQ+ Affirming Care in Healthcare Systems.

Theme	Actionable Items	Expected Outcomes
LGBTQ+ Affirming Care	- Implement training programs focused on attentive listening and equitable treatment. - Develop protocols for recognizing and addressing unique needs, including gender-affirming care. - Encourage providers to advocate for necessary resources and treatments for LGBTQ+ patients.	- Improved patient satisfaction and trust. - Reduced disparities in care quality and access. - Strengthened provider-patient relationships.
Importance of Knowledge/ Training	- Implement comprehensive LGBTQ+ health and identity training for healthcare staff. - Promote active engagement with the LGBTQ+ community, such as participating in community events and continuing education.	- Increased provider competence in LGBTQ+ health issues. - Enhanced cultural competency and allyship among healthcare staff.
Negative Language and Communication	- Train providers to avoid assumptions and use open-ended questions to gather patient information. - Establish guidelines to eliminate repetitive and unprompted questioning that may be perceived as intrusive.	- Enhanced patient comfort and reduced experiences of discomfort or objectification. - Creation of a more respectful and inclusive healthcare environment.
Positive Language and Communication	- Encourage the use of inclusive language, such as “partner” instead of gendered terms. - Train providers to use correct terminology and consistently use patients' pronouns.	- Increased feelings of respect and validation among LGBTQ+ patients. - Strengthened trust and communication between patients and providers.
Inclusive Contextual Cues	- Display visible signs of allyship (e.g. pride flag), and ensure intake forms include options for diverse identities and pronouns. -Train staff to discuss sexual and reproductive health openly and inclusively. -Implement non-discriminatory policies and provide resources for LGBTQ+ care.	- Enhanced sense of safety and belonging for LGBTQ+ patients. - Encouragement of open discussions about sexual and reproductive health needs.

## Author note

The authors of this manuscript vary in the stage of their academic career, with two current graduate student authors, and one early career researcher and assistant professor. All authors have close ties to the LGBTQ+ community, with one author self-identifying as queer and two identifying as pansexual. All authors identify as White and American. Authors primarily work and were raised in the U.S. Two authors have training and theoretical lenses ranging among social psychology, health services, health promotion, and medicine. One author has training in machine learning, data sciences, and software development with both theoretical and practical applications.

## Funding

This research did not receive any specific grant from funding agencies in the public, commercial, or not-for-profit sectors.

## CRediT authorship contribution statement

**Michelle A. Stage:** Writing – review & editing, Writing – original draft, Visualization, Validation, Supervision, Software, Methodology, Investigation, Formal analysis, Data curation, Conceptualization. **Mackenzie M. Creamer:** Writing – review & editing, Writing – original draft, Visualization, Validation, Methodology, Formal analysis, Data curation, Conceptualization, Software, Investigation, Supervision. **Mollie A. Ruben:** Writing – review & editing, Writing – original draft, Validation, Methodology, Investigation, Formal analysis, Data curation, Conceptualization.

## Declaration of competing interest

All authors have nothing to declare.

## Footnotes

2 Percentages were calculated by the number of codes organized under one of the five themes, divided by the total number of codes given across all participants.

## Footnotes

1 https://chatgpt.com/g/g-Tiaz2EQMQ-lgbtq-thematic-analysis
